# Assessment of injection safety in Ha Dong General Hospital, Hanoi, in 2012

**DOI:** 10.12688/f1000research.11399.4

**Published:** 2017-11-24

**Authors:** Phan Van Tuong, Tran Thi Minh Phuong, Bui Thi My Anh, Trang Huyen Thi Nguyen

**Affiliations:** 1 Department of Hospital Management, Health Management Training Institute, Hanoi University of Public Health, Hanoi, 100000, Vietnam; 2 Hanoi Health Administration Bureau, Hanoi, 100000, Vietnam; 3 Hanoi Medical University, Hanoi, 1000, Vietnam; 4 Institute for Global Health Innovations, Duy Tan University, Da Nang, 550000, Vietnam

**Keywords:** Injections, safe injections, injection safety

## Abstract

**Background**: Injection is one of the most frequently used medical methods to introduce drugs or other substances into the body for purposes of treatment or prevention. Unsafe injection can cause adverse outcomes, such as abscess and anaphylactic shock, and increases the risk of blood-borne transmission of viruses to patients and health care workers, as well as the community. Recognizing the importance of injection safety, in 2000 the Vietnamese Ministry of Health (MOH) collaborated with the Vietnam Nurses Association to launch the “Safe injection” program throughout the country, including Hanoi.

**Methods**: This cross-sectional study, combining quantitative and qualitative analysis, was conducted from February to August 2012 in Ha Dong General Hospital using a structured questionnaire and observation checklist. The target population of the study was 109 nurses working in clinical departments and 436 injections were observed.

**Results**: The percentage of nurses who are familiar with injection safety standards was found to be 82.6%. The proportion of practical injections that met the 23 standards of injection safety set by the MOH amounted to 22.2%. The factors related to safe injection practice of nurses who were younger age group (OR=3.1; p<0.05) and fewer number of years working as a nurse (OR=2.8; p<0.05).

**Conclusions**: While nurses have high level of knowledge about safe injections but a small proportion actually practiced. Experience may not always guarantee safe practices.  Injection safety training should be regularly imparted upon all categories of nurses.

## Introduction

Injection plays an important role in medical treatment at hospitals and other medical institutions, especially those where many patients with serious health conditions are treated ^1^. In terms of preventive medicine, vaccination has a significant impact on reducing the incidence and mortality of infectious diseases, which can be prevented by children’s vaccination ^2^.

Despite such positive outcomes, injection can also cause risk of abscess at the site of injection, nerve paralysis, allergic reaction, and anaphylaxis, and, in particular, the risks of transmission of blood-borne viruses to patients, healthcare workers (HCWs) and the community ^3, 4^. According to the World Health Organization (WHO), unsafe injection has become a very common issue and is practiced in many countries; it is the major cause of transmission of diseases such as hepatitis B, hepatitis C and HIV ^2, 5– 7^. The WHO estimates that 50% of injections performed in developing countries are unsafe, and that as many as 20–80% of cases of hepatitis B virus infections are caused by unsafe injections ^2, 5^.

In Vietnam, realizing the importance of safe injection and the risks caused by unsafe injections, in 2000, the Ministry of Health, in collaboration with the Vietnam Nurses Association, launched and implemented the “Safe injection” program across the country ^8^. However, results from some studies after this program was launched show that the rate of injections complying with adequate injection safety standards are not high enough, ranging from 6.0 to 22.6% ^8– 13^. This leading causes related to this low rate of safe injections are: nurses working in understaffed conditions, updated information on injection safety not being conveyed to nurses, non-compliance of technical procedures, and poor infection control operations in injection practices and sample handling, as well as poor management of sharp medical wastes ^14, 15^.

Ha Dong General Hospital, a level I hospital in Hanoi, with a capacity of 550 beds, including 33 departments and specialties, is responsible for the health care of people in the western part of Hanoi city. Following social development trends, the hospital always invests in quality improvement and advancement, including the “Safe injection” program launched by the Vietnam Nurses Association. To provide a description of the situation regarding injection safety in Ha Dong General Hospital, we have conducted a study with the following objectives: (1) Describing the status of injection safety in the hospital; (2) Describing the status of knowledge and injection safety practice of nurses working in the hospital; and (3) Identifying the factors related to nurses’ knowledge and safe injection practice.

## Methods

### Study setting and design

A cross-sectional study was conducted from February to August 2012 in Ha Dong General Hospital, Hanoi.

### Sample and sampling method

The required sample size was calculated based on the WHO manual for sample size determination ( http://apps.who.int/iris/handle/10665/40062). Applying the formula for calculating the one-ratio sample size where the expected rate of safe injections for Ha Dong General hospital was 51.2% (based on a previous study ^4^), confidence level = 95%; and margin of error = 0.05; the minimum sample size was 384 injections. An additional sample size of 10% was added to the minimum sample size to avoid observation failure, resulting in the final sample size = 422 injections. All 109 nurses of the hospital were involved in the administering of injections. Therefore, the number of injections observed for each nurse was 422 /109 = 3.87, which was rounded up to 4 injections per nurse. Therefore, the total number of injections to be observed was 109 × 4 = 436 injections.

The selection of target objects for in-depth interviews and focus group discussions ( Supplementary File 1): 2 in-depth interviews with leaders (Director and hospital Chief Nurse); 14 in-depth interviews with injection performing nurses (randomly recruited); 4 discussion focus groups with the participation of 4 to 6 chief nurses from treatment departments.

### Data collection and measurements

For quantitative research, we used a structured questionnaire with an observation checklist ( Supplementary File 2 and Supplementary File 3) to collect data from 109 participants. Only one observer observed one nurse at one time. The observers were the chief nurses of this hospital. Meanwhile, we conducted in-depth interviews and focus group discussions about key topics, which included work intensity; equipment and instruments; financial factors; forms of reward and encouragement; risk and risk management in injection practices; and other factors affecting nurses’ practices of injection safety.

### Data processing and analysis

Data was encrypted, entered into Epidata 3.0 software, and analyzed using SPSS 16.0 software. Frequency and percentage were used to describe the quantitative data. Chi-squared was used to measure the differences between variables. Odd ratios were calculated to identify the factors associated with safety injection practice. Regarding qualitative data, content transcription from the in-depth interviews and focus discussion groups were categorized into the following topics: workload, equipment, financial factor, incentive and judgement; risk management in safety injection.

### Ethical approval

The study was approved by the IRB of Hanoi University of Public Health (No 029/2012/YTCC-HD3). Data collection procedures and the use of data for analysis were also approved by the directors of the Ha Dong General Hospital. Participants were asked to give written informed consent. They could withdraw from the study anytime without effects on their work or their benefits. Since we observed the regular tasks of the nurse, no informed consent was required from the patients.

## Results

### The injection situation in Ha Dong General Hospital

**Figure 1.**
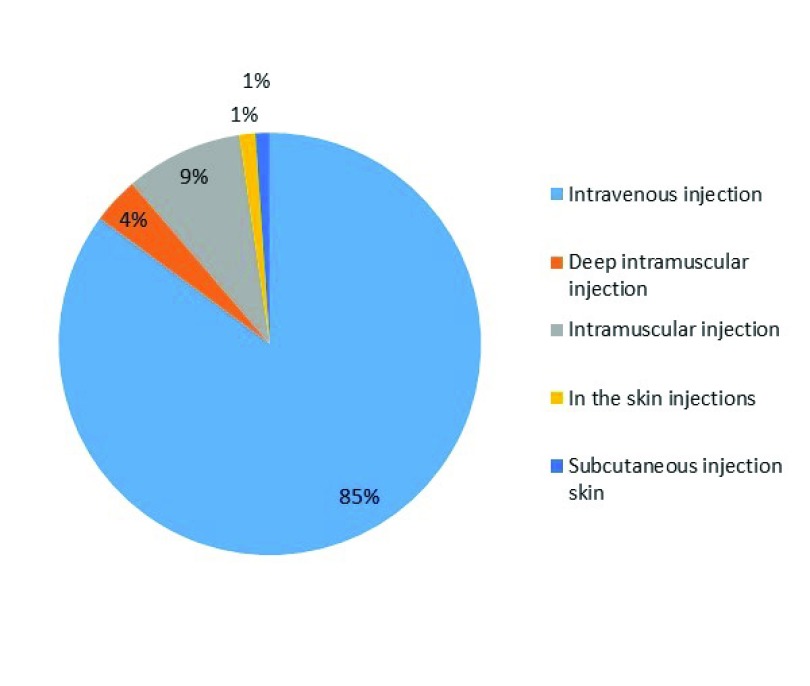
Number of injections by parenteral administration.

Figure 1 shows that 85.1% were intravenous injections; deep intramuscular injections accounted for 3.6%; and only 1.1% were subcutaneous injections, which were usually used for antibiotics testing.

**Figure 2.**
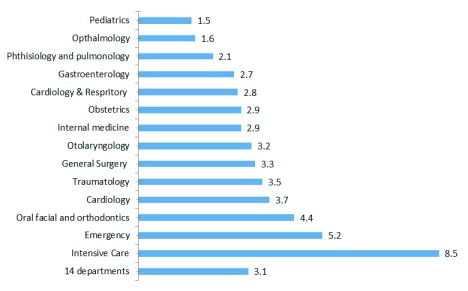
The average number of injections / patient / day by Department.

Injection rate in gluteal muscles accounted for 0.2% ( Figure 2). In the in-depth interviews, where injection-performing nurses were interviewed about why gluteal muscles injections only accounted for 0.2% of the 3.6% of deep intramuscular injections, it was said that *“using deep intramuscular gluteal muscles causes less pain for the patient, but both patient and staff are reluctant to use this method due to cultural reasons” (In-depth interview)*. As for the time of injection, among the 436 observations, the majority of injections were performed in the morning (62.6%) and 7.3% of injections performed in the evening; other injections were performed in the afternoon. In average, each patient received 3.1 injections.

### The situation of nurses’ injuries from sharp objects related to injection

Of the total target population, 41 nurses, accounting for 37.6% of the target population, had been injured by sharp objects, including 36.6% who had been injured 2-3 times. It was mainly caused by performing the wrong injection procedure (75.6%), or due to the unexpected movement of the patient (17.1%), and negligence *(7.*3%). The majority of injuries were to the fingers, accounting for 97.6% of injuries. Regarding the time of day, most injuries happen in the morning (68.3%) followed by the evening (14.6%) and the afternoon (9.8%).

### Demographic of the nursing population

Of the 109 nurses observed in the study, men accounted for 12.8%. The professional qualification of the majority of the nurses in this study was secondary level graduate (83.5%); the average age of nurses in the study was 38.4 ± 11.7 years. Among the overall nursing population, 75.2% were nurses, 22.9% were midwives, and only 1.8% were technicians. The proportion of young nurses working for 5 years or less accounted for 25.7%; 30.3% had more than 25 years of service.

### Current status of nurses’ knowledge regarding providing safe injection

The results in Table 1 shows that 91.7% received training on safe injection in the past year. Up to 26% received training twice in the past year. Most were trained in hospitals (75.2%), only 11% participated in training courses at the Provincial Health Offices, and 8.3% had never been trained in injection safety. In addition to the general training program, the chief nurse often provided guidance on safe injection practices and knowledge for the nurses at the hospital (98.2%). The majority of nurses (95.4%) knew that in treatment rooms of departments, materials on injection safety are readily available.

**Table 1.** The situation of providing safe injection knowledge for nurses.

Knowledge provided on safe injection (n=109)	Frequency (n)	Percentage (%)
**Training courses on safe** **injection in the past year**		
Yes	100	91.7
*1 time*	90	82.6
*2 times*	8	7.3
*3 times*	2	1.8
No	9	8.3
**Unit responsible for the** **majority of training courses**		
Provincial Health Office	12	11
Hospital	82	75.2
Provincial health Bureau & Hospital	7	6.4
**Chief nurse provides** **guidance currently**		
Yes	107	98.2
No	2	1.8
**Documents on safe injection** **available in the department**		
Yes	104	95.4
No	5	4.6

### Nurses’ knowledge of safe injection techniques

Table 2 shows that the proportion of nurses having good knowledge (≥17/21 right answers; <17/21 right answers – insufficient knowledge) in injection safety was 82.6%, but there were only 1 in 23 questions in which 100% nurses gave the correct response, of which content involved checking the quality of drugs before injection. There were 4 departments where some nurses gave 100% correct responses: emergency, ophthalmology, internal gastrointestinal, and internal cardiovascular departments. 

**Table 2.** Knowledge of injection safety of nurses by department.

No	Department	Knowledge of safe injection					
		Insufficient		Sufficient		Total	
		n	%	n	%	n	%
1	Cardiovascular respiratory	1	33.3	2	66.7	3	2.8
2	Pediatrics	2	20.0	8	80.0	10	9.2
3	General external	2	20.0	8	80.0	10	9.2
4	Ophthalmology	0	0.0	5	100.0	5	4.6
5	Tuberculosis	1	20.0	4	80.0	5	4.6
6	Ears nose and throat (ENT)	1	33.3	2	66.7	3	2.8
7	General internal	2	22.2	7	77.8	9	8.3
8	Intensive care	1	10.0	9	90.0	10	9.2
9	Obstetrics	4	20.0	16	80.0	20	18.3
10	Internal gastrointestinal	0	0.0	8	100.0	8	7.3
11	Trauma	2	25.0	6	75.0	8	7.3
12	Odonto-stomatology	3	75.0	1	25.0	4	3.7
13	Internal cardiovascular	0	0.0	5	100.0	5	4.6
14	Emergency	0	0.0	9	100.0	9	8.3
	**Total**	**19**	**17.4**	**90**	**82.6**	**109**	**100**

### Safe injection practices at Ha Dong General Hospital

***Practice in the preparation of materials and injection devices*.** About 99.1% injections were fully prepared with a box for dealing shock during injections on the trolley, 97.7% with sharp object containers and hand antiseptic in a convenient location on the injection trolley, 94.0% had sterile needles and syringes. Yet, 6% of injections were made when nurses had not checked the integrity of the needle packaging, and without injection trolley and equipment (2.1%). 86.5% of observed injections achieved all five main criteria.

***Practice of aseptic principles in the administration of injections.*** Table 3 shows that the rate of injections performed in which nurses had cleaned their hands before administration was 63.1%. In 17% of injections, needles remained on the bottles, and 20% of injections were performed when no antiseptic techniques were applied to the medication containers, or with unchecked needles. The rate of injections performed by nurses in compliance with 4 sterile criteria in the injection process was only 45.0%.

**Table 3.** Percentage of compliance with aseptic principles of injection safety, as collected via observation.

Aseptic principles of safe injection	Number of injection observed (n=436)	
	n	%
**Wash hands/fast hand disinfection** **before injection**		
Yes	275	63.1
No	161	36.9
**Sterile when taken drugs before** **injection**		
Yes	349	80.0
No	87	20.0
**Injection needle kept on the bottle** **after taking the drug**		
Yes	362	83.0
No	74	17.0
**Ensure sterile injection needle**		
Yes	349	80.0
No	87	20.0
**Ensure the principle of sterile** **needles (at 4/4 standard)**		
Yes	196	45.0
No	240	55.0

***Practice of safe injection techniques.*** In 97.9% of injections, the nurses identified the injection sites correctly, 83% of injections were performed in compliance with the 5 standard techniques (identify correct injection position, sterilize the skin before injection, check quality of drug, perform correct injection technique, and sterilize the skin after injection) and the rate of quality control of medicine was 85.1%. The rate of proper skin disinfection before injection was 91.1%, but only 81% complied with standard disinfection practice immediately after injection. The rate of injections complying with technical criteria of injection was 66.5%.

***Interactive communication with the patients.*** Via observation, the rate of injections in compliance with the 5-correct injection techniques (correct patient, correct drug, correct dose, correct injection way, correct time) was 100%, the rate of maintaining care records and medical order books was 93.3%, and the lowest rate was the communication and observation of patients while performing injections, especially interactive communication with patients after injection, which was only 67.7%. Results of in-depth interviews also showed that communication with patients while conducting injection was not sufficient; before injecting, most nurses performed observation, gave guidance and prepared for the injection, but communication during and after injection was given incompletely or superficially, *“their way of communication did not show any enthusiasm, or sympathetic and sharing attitude, and without motivation or encouragement to patients for their cooperation in the performance of injection”* - (Focus group discussion)

***Practice of prevention of infection risks for patients and the community.*** 46.1% of the injections were performed in compliance with all the four technical standards (wear glove when intravenous, did not use hands to remove the needle, isolate syringes and needles, hand wash after injection) to prevent risks for people receiving injections and the community. Similar to the rate of hand disinfection before injection, only 61.9% performed quick hand disinfection after injection. 68.1% wore hand gloves when administering intravenous injection. It was reported by nurses in in-depth interviews that *“it is difficult to perform intravenous injection if gloves are worn, especially in providing injections to small children”*- (HCWs-PVS). The rate of nurses using their bare hands in covering and removing needle caps was 88.8%. The highest rate was the rate of injections in compliance with the provision of isolating needles immediately after injection, which was 93.3%.

***Practical injections meeting safety standards criteria.*** The rates of injections that meet the safety injection (SI) criteria (correct preparation of injection equipment, ensure sterility requirement, correct injection technique, correct communication with patients, prevent risk for people receiving injection and ensure the safety standard of injection) at different departments ranged from 11.1% to 33.3%, with the lowest in the Emergency Department, with only 4 in 36 injections (8.3%), the rate in the Odonto-stomatology Department was 12.5%, and the highest was in the Pediatrics Department, with 14/42 injections, accounting for 33.3%.

Table 4 shows that there are six nurses who do not have any of the four observed injections in compliance with the SI standards (5.5%). Only 9/109 nurses had all four observed injections meeting the criteria of injection safety (8.3%). There were 26 nurses who have at least three injections meeting the required standards (26.9%).

**Table 4.** Percentage of nurses having correct safe injection procedure practice.

SI practice of the nurse (n=109)	No. of injections meeting required standards	Frequency (n)	Percentage (%)
Practice properly	4/4	9	8.3
	3/4	17	15.6
Not meet standards	2/4	24	22.0
	1/4	53	48.6
	0/4	6	5.5
**Total**		**109**	**100**

### Factors related to the nurses’ knowledge and practice of injection safety

***Factors related to safe injection.*** The rate of nurses with knowledge of SI in the nurses aged up to 30 years was 93.2%, 3.3 times higher than those aged over 30 years (75.4%) (OR = 4.4; p <0.05). No statically significant difference was found in the rate of nurses having knowledge of good SI between nurses of different genders and different levels of education (p> 0.05).

Among nurses with sufficient SI knowledge, the number of nurses with <10 years of work was 4.9 times higher than that of nurses with work experience of >10 years (OR = 4.9; p <0.05). The rate of nurses with sufficient SI knowledge among nurses who received training in the past year was 86.1%, which was 10.3 times higher than the untrained group. This difference was statistically significant (p <0.001). However, there was no statically significant difference in the rates of sufficient knowledge of SI and the different levels of nurses (for example: college nurses or university nurses) (p>0.05).

The rate of injury due to sharp objects during injections in the group with no knowledge of SI was 63.2%, which is 3.6 times higher compared to the group with knowledge about SI (32.2%). The difference was statistically significant (X ^2^ = 6.39; p <0.05).

***Factors related to safe injection practice.*** Age was a statistically significant factor with regard to safe injection practices (X ^2^ = 6.3, p <0.05), the rate of correct practice of SI in nurses <30 years was found to be 3.1 times higher than those >30 years old.

Table 5 shows the percentage of correct practices in the nursing team with work experience of <10 years (32.7%); 2.5 times higher than the senior group (work experience >10 years) (14.8%). This difference was statistically significant (p <0.05). Professional qualifications and the number of injections / day of each nurse had no statically significant association with SI practice (p> 0.05). The rate of correct practice of the group with knowledge about SI (26.7%) was three times higher than the group with no knowledge (10.5%); this difference was not statistically significant (OR = 3, 09; p> 0.05).

**Table 5.** Relationship between qualifications, seniority, number of injections per day and SI practice of nurse.

Characteristics	Safety injection practice (n=109)				Validation value		
	Incorrect		Correct		OR	95% CI	p
	n	%	n	%			
**Qualification**							
Other	22	81.5	5	18.5	1.5	0.5 – 4.5	0.45
Nursing	61	74.4	21	25.6			
**Seniority, years**							
≥ 10	46	85.2	8	14.8	2.8	1.1 – 7.2	0.028
< 10	37	67.3	18	32.7			
**Number of** **injections per day**							
≤ 10	42	71.2	17	28,8	0.5	0.2 – 1.3	0.18
>10	41	82.0	9	18.0			
**Age group, years**							
> 30	55	84.6	10	15.4	**3.1**	**1.3 – 7.8**	**0.012**
≤ 30	28	63.6	16	36.4			
**Gender**							
Male	11	78.6	3	21.4	1.1	0.3 – 4.6	0.82
Female	72	75.8	23	24.2			
**Education**							
Vocational training	72	79.1	19	20.9	2.4	0.8 – 7.1	0.1
College/University	11	61.1	7	38.9			

### Factors related to injection safety in Ha Dong General Hospital

Of the 436 observed injections, 273 injections were observed in the morning (62.6%). Safe injection rate in the morning was 22%. The rate of injection safety at midday was the highest (88.9%); in the afternoon, 95 injections were observed, but no injections were found to meet all the 23 criteria of SI. There were statically significant differences regarding injection safety depending on the time of the day at which injections are applied, which the highest percentage of safety injection was at noon (X ^2^ = 120.4; p <0.001).

Intravenous injections were observed at the highest rate (62.4%), but only 19.1% were found to be meeting SI standards. Safe injection rate of the observed subcutaneous injections was 57.1%, and for injection in the skin, this was 33.3%. The difference in the rate of safe injection according to injection type was statistically significant (X ^2^ = 23.4; p <0.001). The number of intravenous injections was the most directly observed, with 173 injections out of 436 injections observed (50.5%), but the safety injection rate was only 21.4%. The safe injection rate was lowest in the intravenous injections via fork / rubber joints (9.3%) and the highest intramuscular injections in the thigh quadriceps (60%). However, this difference was not statistically significant (p> 0.05).

The first observed injections had the highest safety rate of 58.7%. Safe injection rate of the observed second injections was 7.3%, 3rd was 10.0% and the fourth was 13.0%. This difference was statistically significant; (X ^2^ = 112.7; p<0.001).

The results of the in-depth interviews show the cause of unsafe injection. One reason was mentioned is that nurses were overloaded their work: *“In the morning I have to injecting [sic] dozens of patients, so how can [I] follow the process!”*. With this workload, they felt stressful and therefore, they could not follow the procedure of safe injection practice. Additionally, some nurses injected as their habits with old procedures, which did not ensure the safe injection practice: *“Nurses with high age are very fluent in the use of fluids but often follow the old procedures, often bypassing, cutting down the process, changing the way they are” (In-depth interview).* Another reason also mentioned is the regular supervision of the chief nurse “ *If the chief nurse regular[ly] supervises the injection procedure, the nurses will mandatorily follow the procedure of safe injection practice*”.

Raw data obtained from the questionnaire assessing knowledge for safe injection practice among nurses Click here for additional data file. Copyright: © 2017 Van Tuong P et al. 2017 Data associated with the article are available under the terms of the Creative Commons Zero "No rights reserved" data waiver (CC0 1.0 Public domain dedication).

Raw data obtained from the observation assessing practice for safe injection practice among nurses Click here for additional data file. Copyright: © 2017 Van Tuong P et al. 2017 Data associated with the article are available under the terms of the Creative Commons Zero "No rights reserved" data waiver (CC0 1.0 Public domain dedication).

## Discussion

This study provided baseline evidence for further interventions to improve safe injection practice in Vietnam. In this study, we found that while nurses have high level of knowledge about safe injections but a small proportion actually practiced. Moreover, regression results indicated that experience may not always guarantee safe practices.

This research showed that on average each patient received 3.1 injections. Compared to the results study in other countries, this rate is lower than result study of HAURI Global 2000 study ^16^. However, this result is higher than Tu’s study ^17, 18^ and research by the Vietnam Nurses Association in 2010 ^14, 19, 20^. About 37.6% nurses had been injured by sharp objects. The sharp injury rate at Ha Dong Hospital is higher than the results of Muc’s study ^14^, Nguyen Tu’s 2005 study ^17^, and lower than that of the Vong *et al*’s study in Cambodia ^21^. Most of the injuries occurred in the morning. Unintended activities is the cause of most injuries. Meanwhile, sharp instruments injuries accounted for most of the fingers wounds.

### Knowledge and practice of injection safety among nurses Ha Dong General Hospital

***Knowledge about safe injection standards of nurses.*** The ratio of nurses having knowledge about safe injection was found to be higher in the present study compared with the study of Ernest *et al*. at City Hospital Benin Nigeria ^22^. These rates are lower than those of Phan Canh Chuong at the Hue Central Hospital ^10^, but higher than that found in a previous study by Thanh ^8^.

Nearly half of injections followed the 4 sterile standards. For example, 50% of injections followed regulation on communication standards, and most injections followed proper safety standards for injected persons; however, 17% and 32% of injections did not isolate the needle and syringe immediately after injection and only 32% used gloves when injecting intravenously.

### Factors related to knowledge and practice of safe injection in Ha Dong General Hospital

Nurses aged up to 30 years had better knowledge and higher rate of safe injection practices than nurses >30 years old. Nurses with less than 10 years of work experience had better knowledge level and higher safe injection practices than senior nurses with 10 years or more experience. Regarding training, nurses trained for 1 year had better knowledge than untrained groups, and as a result untrained groups were more likely to be exposed to accidental injuries than that of knowledgeable groups. These findings were similar to other previous studies ^9– 11^. The rate of correct practice of the group with sufficient knowledge was higher than the group with insufficient knowledge, but this difference was not statistically significant (p > 0.05), which was consistent with other studies ^8, 17, 20^. The results of the in-depth interviews also showed that old habits, e.g. bypassing the injection process, and the supervisor’s supervision were also influential. Meanwhile, injection timing, parenteral administration and order injections were observed factors that have statistically significant relationship (p < 0.05) with the rate of safe injections of the hospital.

A number of recommendations can be made based on the results of the study: (1) Enhancing the sterilization performance, reducing the risk of infection due to injury; (2) Promoting training courses to improve knowledge and skills, educational communication to increase knowledge and awareness of risk of injections; (3) Establishing a regular injection safety monitoring and assessment program - the results and related information must be reported to management and disseminated to hospital staff; (4) Enhancing the inspection and supervision of regimes of reward and sanction, of emulation and commendation, and conducting research of SI assessment; (5) Focusing on the principles of sterilization, hand hygiene before injection, sterilization routine when taking drugs, and sterilization of needles in injection safety training, as well as on enhancing communication skills in dealing with patients.

## Conclusion

Despite the high level of knowledge about safety injection, a low proportion of nurses performed correct safety injection practice. Moreover, the results demonstrated that experience might not always guarantee better practice. The findings raise the need for further training about this issue, especially among older nurses.

## Data availability

The data referenced by this article are under copyright with the following copyright statement: Copyright: © 2017 Van Tuong P et al.

Data associated with the article are available under the terms of the Creative Commons Zero "No rights reserved" data waiver (CC0 1.0 Public domain dedication). 

Dataset 1: Raw data obtained from the questionnaire assessing knowledge for safe injection practice among nurses. doi, 10.5256/f1000research.11399.d165390 ^23^

Dataset 2: Raw data obtained from the observation assessing practice for safe injection practice among nurses. doi, 10.5256/f1000research.11399.d165391 ^24^

The transcripts of the in-depth-interview and focus group discussion are not available due to the sensitive information contained. However, this information will be made available for university researchers who send a request to Prof. Tuong Van Pham, PI of the study: pvt@huph.edu.vn.

## Supplementary material

Supplementary File 1: In-depth interview guide and group discussion guide.

Supplementary File 2: Questionnaire assessing knowledge for safe injection practice among nurses.

Supplementary File 3: Observation checklist assessing practice for safe injection practice among nurses.
