# Supplementary material for: Assessment of injection safety in Ha Dong General Hospital, Hanoi, in 2012
Source: F1000Res. 2017 Nov 24;6:1003. Originally published 2017 Jun 26. [Version 4] doi: 10.12688/f1000research.11399.4 (PMC5698916; doi:10.12688/f1000research.11399.4)
Supplement: Supplementary file 4 [file f1000research-6-14401-s0003.tgz › 4fc96d95-0316-4aa7-872c-9b3c2e1e7449.docx]

**SAFETY INJECTION SURVEILLED**

**(For Nurses / Midwives / Technicians)**

This questionnaire is aimed at assessing the current situation of injections at health facilities. We do not evaluate individuals and entities providing information in this survey. Would you please collaborate by providing enough information in the form. Your information helps the research team assess the safety of injection and thereby improve the quality of care and treatment. patient.

No. : …………………

**Department**:

1. Cardiology and Respiratory
2. Pediatric
3. Surgery
4. Ophthalmology
5. Pathobiology and pulmonology
6. Otolaryngology
7. Internal medicine
8. Intensive care
9. Obstetric
10. Gastroenterology
11. Traumatology
12. Oral facial and orthodontics
13. Cardiology
14. Emergency

Date …….month……..year…….

**A. General Information:**

A1k. Year of birth: .....................

A2. Gender: ..................... 1. male                 2. Female

A3k. Education: 1. Beginner               4. University

                                                        2. Intermediate 5. Postgraduate

                                                        3. College 6. Other (specify) ..................

A4k. Qualification: 1. Nursing 2. Midwife 3. Technician

A5k. Place of professional training: ..................................................................

A6k. Years of service: ........................................................................... ..

A7k. Number of injections perform per day: ....................................................

**B. Information on training, providing safe injection knowledge**

B1k. Number of times training / Safety injection training in the past year: ............... .. times

B2k. You attended the training organized by any organization:

1. Hospital                             2. Department of Health                             3. Other: ..................

B3k. Number of times training / training on safety injection during work ... time

B4k. You attended as a representative of:

1. Hospital                             2. Department of Health                             3. Other: ..................

B5k. Under the guidance of Head of Department of safety injection knowledge and practice:                                           1  . Yes                             2. No.

B6k. Under the guidance of Head of Department of safety injection knowledge and practice:                                           1 . Yes                             2. No.

B7k. Is the safety injection manual available, easy to find in the department / department?

1. Yes               2. No.

**C. Knowledge about safe injection**

           Here are some multiple choice questions about safe injection. He / she please mark X in **column 01** corresponding to the answer he / she.

| **No.** | **Circumstance** | **(1)**  **Right** | **(0)**  **Wrong** |
| --- | --- | --- | --- |
| C1k | People who hold used injection equipment are at risk of infection and injury. |  |  |
| C2k | Hepatitis B virus is not transmitted through needles |  |  |
| C3k | Good Practice: Hold gauze pads in the bleeding area of ​​the injection site |  |  |
| C4k | There is no need to wash hands between shots. |  |  |
| C5k | Single-use injectable syringes can be sterilized and re-used. |  |  |
| C6k | Do not cover, bend or break a needle before breaking. |  |  |
| C7k | Do not remove needles from the syringe by hand. |  |  |
| C8k | One-time sharps containers should be covered, sealed, labeled at full ¾ |  |  |
| C9k | Burning is the safest way to destroy sharp containers. |  |  |
| C10k | Burning at high temperatures can help to reduce air pollution over low-temperature combustion. |  |  |
| C11k | It is acceptable to cover needles when you have to transport needles to other sharp containers (because the barrel you are using is full). |  |  |
| C12k | Syringe vaccines are less likely to be re-used. |  |  |
| C13k | Never leave the needle in the medicine cover to withdraw the medication several times. |  |  |
| C14k | If a patient wants to receive a shot in order to feel healthier, khe / she should give the injection (to achieve a false-positive effect). |  |  |
| C15k | Injections are always more effective than taking pills. |  |  |
| C16k | Injecting the injection site can be harmful to the person injected |  |  |
| C17k | There is no need to check the quality of the drug before injection |  |  |

C18k. How can we use the following to clean the injection site?

1. There is no need to clean the injection site
2. Can be wiped with 70% alcohol, Iod alcohol 1%
3. Use only water.
4. One of two ways b, c above is.

C19k. Proper disposal of injection supplies and other sharp objects intended to:

1. Minimize infectious diseases.
2. Reduce the risk of sharp injury.
3. Improving the medical facility landscape.
4. All purposes above.

C20. Destruction properly needles and sharp objects were used, including: ***(Multiple choice)***

C20a Always break or bend a needle before disposal to prevent reuse.

C20b Put needles and syringes attached to the puncture boxes / barrels.

C20c Only move to destroy the crates filled with sharp objects.

C20d Separate the needle from the syringe with two hands.

C20e Do not use any way above.

C21. Unsafe Injections increase the spread of blood-borne diseases can be overcome by: ***(select multiple choices)***

C21a Minimize unnecessary injections.

C21b Burn the syringe completely to the point of destruction.

C21c Use only sterile syringe to vaccinate.

C21d Three above

**D. Safe injection in hospital**

D1 What is the rate of infusions that pass the criteria for safety injection assessment at the hospital where you work? ..................%

D2 Causes nursing noncompliance safe injection process are: ***(select multiple choices)***

D2a Lack of safety injection knowledge

D2b Medical equipment is inadequate and inadequate

D2c Lack of supervision

D2d Lack of penalties

D2e There is no emulation movement

D2f High intensity / overload

D2g All above

D2h Other (specify) ..................................................................... ..

3. Main cause of insecurity is due to the following factors: ***(select multiple choices)***

              D3a The patient / family member likes to be given a shot rather than taken

              D3b Specify over-infusion required

              D3c Practice injection technique of medical staff

              D3d Lack of medical equipment needed to implement safety injection

              D3e Lack of means to collect sharp objects

              D3f Sharp objects are not completely destroyed

              D3g. All above

D3h. Other (specify) .........................................................

**E. Prevention of infection and occupational accidents**

E1. From 6/2011, were you injured by sharp objects?

              1. Yes

              2. No 🡪 **Go to section 7 of this section**

E2. Risk: ................. times

E3. Location of injury:

1. finger 2. hand 3. other: ........................ ..

E4. What are the causes of most of the sharp injuries? ***(Choose one)***

              1. Due to negligence

              2. The patient leaps

              3. Unknowingly

              4. Other (specify) ..................................................................... ...

E5. When you were injured by the sharp object?

1. Morning               2. afternoon 3. evening

**SINCERELY THANK!**
