# Supplementary material for: Assessment of injection safety in Ha Dong General Hospital, Hanoi, in 2012
Source: F1000Res. 2017 Nov 24;6:1003. Originally published 2017 Jun 26. [Version 4] doi: 10.12688/f1000research.11399.4 (PMC5698916; doi:10.12688/f1000research.11399.4)
Supplement: Supplementary file 5 [file f1000research-6-14401-s0004.tgz › cb071dde-6d83-4dd9-b2fb-c668b52e0a64.docx]

SAFETY INJECTION ASSESSMENT SHEET

Hospital:…………………………… Assessment sheet No.: ……………

Hospital department:

1. Cardiology and Respiratory
2. Pediatric
3. Surgery
4. Ophthalmology
5. Pathobiology and pulmonology
6. Otolaryngology
7. Internal medicine
8. Intensive care
9. Obstetric
10. Gastroenterology
11. Traumatology
12. Oral facial and orthodontics
13. Cardiology
14. Emergency

Observation time: ………….hour……… day ……….month………..year……….

Name of health worker who injected :……………………………………………….

Name of observer:…………………………………………………………………….

**A- General information**

a1. Patient’s name: …………………………………………………………………..

a2. Patient’s age: …………………………………………………………………..

| **A3. Injection time:**   1. Morning 2. Noon 3. Afternoon 4. Night | **A4. Router of administration**:   1. Intravenous injection; 2. Deep intramuscular injection; 3. intramuscular injection; 4. In the skin injections; 5. Subcutaneous injection | **A5. Injection location:**   1. Delta Mechanical; 2. Quadriceps thigh; 3. Arm trilum; 4. Hoofed; 5. Inject the veins through the triple heel / rubber joint; 6. Direct intravenous injection 7. Other …………… |
| --- | --- | --- |

**B- Safety Injection Assessment**

| **No.** | **Criteria** | **(1)**  **Yes/Right** | **(0)**  **No/Wrong** | **Note** |
| --- | --- | --- | --- | --- |
| I. | **Preparation of injection equipment and tools** | |  |  |
| B1 | Sterile syringe needles |  |  |  |
| B2 | Injection vehicles and equipment, tools attached |  |  |  |
| B3 | Shockproof box sufficient number prescribed on the vehicle injection |  |  |  |
| B4 | Quick disinfectant hand in convenient position |  |  |  |
| B5 | Sharp box enough, suitable |  |  |  |
| **II.** | **Practice injection procedures** | |  |  |
| B6 | Hand wash / disinfect hands before injection |  |  |  |
| B7 | Ensure sterile needles |  |  |  |
| B8 | Sterilize when taking medicine before injection |  |  |  |
| B9 | Antiseptic properly regulated injection site before injection with antiseptic solution (alcohol 70 ^0,^ 1% iodine alcohol) |  |  |  |
| B10 | The injection site identifies the injection site |  |  |  |
| B11 | Wear gloves |  |  |  |
| B12 | Perform the following actions: (B121) evacuate; (B122) Right angle of the needle: In the skin 10-15 degrees; Under the skin 25-30 degrees; Corn shallow 45-60 degrees; The depth of 90 degrees, (B123) Depth: Do not plug the needle body; (B124) withdraw piton blood test; (B125) Make 2 fast 1 slow (1ml / 10s) |  |  |  |
| B13 | The injection site uses the correct medication |  |  |  |
|  | (B131) Right patient |  |  |  |
|  | (B132) Right medicine |  |  |  |
|  | (B133) Correct dose |  |  |  |
|  | (B134) Correct router of administration |  |  |  |
|  | (B135) Correct time |  |  |  |
| B14 | Examination of drug quality by sensory (color, shelf life, ...) |  |  |  |
| B15 | Antiseptic properly regulated injection sites after vaccination with antiseptic solution (alcohol 70 ^0,^ 1% iodine alcohol) |  |  |  |
| B16 | Do not store the needle on the vial after taking the medication |  |  |  |
| B17 | Wear gloves when given intravenously or infused |  |  |  |
| B18 | Do not use hands to remove the needle |  |  |  |
| B19 | Isolate syringes and needles right after injection |  |  |  |
| B20 | Hand wash / disinfect quickly after the injection |  |  |  |
| B21 | Record nursing care records and checkbooks |  |  |  |

Supervisor

*(Sign, write full name)*
