# Supplementary material for: Assessment of injection safety in Ha Dong General Hospital, Hanoi, in 2012
Source: F1000Res. 2017 Nov 24;6:1003. Originally published 2017 Jun 26. [Version 4] doi: 10.12688/f1000research.11399.4 (PMC5698916; doi:10.12688/f1000research.11399.4)
Supplement: Supplementary file 3 [file f1000research-6-14401-s0002.tgz › 98705fe4-2088-4981-99f0-04b454b7ca90.doc]

**In-depth interview guide**

**I. Purpose:** Learn the factors related to safe injection practices

**II.** **Subjects:**

-          Representative of the hospital management

-          Head nursing hospital

**III.** **Interview content:**

1. General comment on infusion work in the hospital?
2. Safety injection ratio in hospital?
3. What factors affect the current knowledge and practice of safety injection for nursing?
4. Cause nursing does not comply with the rules in the safety injection
5. How does the clinic provide incentives, fines, incentives, incentives for individuals and departments to implement good / bad injection procedures?
6. What measures have the hospital taken to ensure safe injection?
7. Comment on the effectiveness of these measures?
8. Suggest solutions to improve the effectiveness of these measures / improve the rate of hospital injections?

**Group discussion guide**

**I. Purpose:** Learn the factors related to safe injection practices.

**II.** **Subjects:**

-          Group nursing department heads / departments

**III.** **Interview content:**

1. General comment on infusion work in the hospital?
2. Safety injection rate at the hospital?
3. What factors affect the current knowledge and practice of safety injection for nursing?
4. What causes nursing not to follow the rules in a safe shot?
5. What factors affect the current practice of safety injection for nursing?
6. Evaluation of the quality and quantity of medical equipment, protective equipment provided?
7. What steps have the hospital taken to ensure safe injection procedures?
8. Comment on the effectiveness of these measures?
9. Suggest solutions to improve the effectiveness of these measures / improve the rate of hospital injections?
