# Supplementary material for: Assessment of injection safety in Ha Dong General Hospital, Hanoi, in 2012
Source: F1000Res. 2017 Nov 24;6:1003. Originally published 2017 Jun 26. [Version 4] doi: 10.12688/f1000research.11399.4 (PMC5698916; doi:10.12688/f1000research.11399.4)
Supplement: Peer review history

# Peer review: Referee response for version 4

**DOI:** 10.5256/f1000research.14401.r27522

**Published:** 2017-12-06

**Authors:** Alexandre Hannud Abdo

**Affiliations:** 1 Laboratoire Interdisciplinaire Sciences Innovations Sociétés (LISIS), Université Paris-Est, Paris, France

This is a mixed methods exploratory study on injection safety and related factors, including training programs, in a hospital in Vietnam. The article is aptly written, though with caveats to be mentioned. Statistical treatment is unsophisticated but sufficient for the kind and aims of observations made in the text. More advanced statistical treatment could potentially reveal further interesting features of the data.

 I felicitate the authors for sharing their work on this important issue, and for having undertaken such work in the first place.

 I would also first ask the authors to look at and respond to the comments by Dr. Sindhu. He makes a series of points that do need either clarification or correction. Some of the discrepancies he mentions, looking at your spreadsheets, might be caused by missing data. But where that is the case it should be reflected in the text, which it currently isn't, leading attentive readers to confusion.

*On language*, the text is of good quality, but there are a few mistakes that ought to be avoided as they may lead to confusing interpretations by unfamiliar readers:

 (a) "unsafe injection (...) is **the** major cause of transmission of diseases such as hepatitis B, hepatitis C and HIV ^"^, one can see the authors mean "is **a** major cause";

 (b) "Data was encrypted", one supposes the authors mean "anonymized";

 (d) "The results of the in-depth interviews show **the** cause of unsafe injection", one supposes the author means "show **some possible** causes of";

*On the data provided*, I could not find the dataset with the 436 observed injections. Both datasets provided contain 109 entries, so both seem to have one entry per nurse. However, the columns of the second dataset seem to refer to the injection assessment questionnaire, which I would expect to have one entry per injection, thus 436 entries. I would ask the authors to either better explain what is showed in this spreadsheet, or provide the full dataset if there was some mistake - or both if this is an intermediate dataset.

 It would also be useful, in case the original questionnaires were applied in Vietnamese, to have copies of those as well, for the sake of future research.

*On the analysis*, given the goal of understanding the impact of training programs, why wasn't the relationship between "trained in the last year" and "correct SI practice" considered? Also, table 5 doesn't show the numbers for "knowledge of SI" and "correct practice" which are mentioned in the text.

 Still on the analysis, when the authors mention "A number of recommendations can be made based on the results of the study...", it seems those 5 recommendations are presented without a specific order or priority, and they are presented disconnected from the previous analysis. First, the lack of an established priority should be made explicit by avoiding numeric items. Other than that, it should be attempted to provide some cue to the reader as to whether a recommendation is supported by the statistical analysis, by the interviews, or both, even if the reader could in principle recover this by going back and forth in the text.

*On the discussion*, given that the account of qualitative interviews emphasizes nurse overload, I would suggest for this study to recommend that future studies include some quantitative account of the level of overload that goes beyond the simple number of injections, as nurses can be overloaded by other issues. I would mention, for one, the literature on burnout syndrome.

I have read this submission. I believe that I have an appropriate level of expertise to confirm that it is of an acceptable scientific standard, however I have significant reservations, as outlined above.

# Peer review: Referee response for version 3

**DOI:** 10.5256/f1000research.13787.r27387

**Published:** 2017-11-21

**Authors:** Arshad Altaf

**Affiliations:** 1 Service Delivery and Safety, World Health Organization, Geneva, Switzerland

Overall the article is good and contributing to a serious health care issue.  It would be really helpful if a native English speaker can review it one time to improve the flow of the language. 

**Abstract**

**Results, 3 ^rd^ sentence: suggestion **The factors that were related to safe injection practices included nurses who were younger OR… and fewer number of years working as a nurse OR…

**Conclusion: **While nurses have high level of knowledge about safe injections but a small proportion actually practiced. Experience may not always guarantee safe practices.  Injection safety training should be regularly imparted upon all categories of nurses.   

**Introduction:**

  

1. Please write full details of reference 1
2. Please delete reference number 5 and use these two references:  1 Pepin et al.  Evolution of the global use of unsafe medical injections, 2000-2010. PLoS One. 2014 Jun 9;9(6):e99677. 2  Pepin et al.  Evolution of the global burden of viral infections from unsafe medical injections, 2000-2010.  PLoS One. 2014 Jun 9;9(6):e99677.  According to the first study the global burden of unsafe injections is 5.5% and the number of infections associated with unsafe injections is mentioned in the second paper.  Please read and update this detail.  

**Results:**

  

1. Current status of nurses knowledge regarding safe injection:  In the table it says 7.3% in front of 2 time? 
2. Nurses knowledge of safe injection technique:  Please reduce the text since tables are quite self explanatory.  Long readings tend to distract the readers
3. Safe injection practices at Ha Dong hospital:  First sentence is a comment.  In the results please present what the study found and refrain from comments in the results section
4. “anti shock injection box” is not a common term.  Suggestion is to please explain it.  
5. Table 4:  Suggestion to remove the column on cumulative percentage as it is not providing any objective information.  It has the potential to confuse the reader. 

 

**Discussion:**

 Please use the first paragraph of discussion to highlight four or five key findings of the study.  In subsequent paras please explain some of these findings in light of other published literature.  If no study is available use your own judgement.

I have read this submission. I believe that I have an appropriate level of expertise to confirm that it is of an acceptable scientific standard.

# Response

**Published:** 2017-11-22

**Authors:** Trang Nguyen

**Affiliations:** Institute for Global Health Innovations, Duy Tan University, Vietnam

Dear Dr.Arshad Altaf,

 Thank you very much for your very constructive comments. We have revised according to your suggestion in the new version.

 Best regards,

 Authors

# Peer review: Referee response for version 1

**DOI:** 10.5256/f1000research.12306.r24673

**Published:** 2017-09-14

**Authors:** Mattias Larsson

**Affiliations:** 1 Department of Public Health Sciences, Karolinska Institutet, Stockholm, Sweden

Thank you for a well written article with an important message - that injection safety can be improved which might decrease injection related disease transmission.

 Comments:

 In Abstract it states:

1. The nurses who are familiar with injection safety standards ... 82.6%
2. The proportion of injections that met the 23 standards of injection safety ... 22.2%.
3. A low proportion of nurses performed correct safety injection...

This indicates a large difference between knowledge and practice. It would also be of use to have the average of the number of safety standards that was recorded. 

 In Table 1. the headline "Training courses on safe injection in the past year " occurs twice with different figures which is not clearly explained in the text. 

 Experience might not always guarantee better practice - "rate of correct practice of SI in nurses <30 years was found to be 3.1 times higher than those >30 years old"

 Clarifications:

1. Question "Are all the source data underlying the results available to ensure full reproducibility?"
  Partly as it would be good to have a shortlist of all 23 standards and how each of them was assessed as well as the result (average, SD, median)
2. Question "Are the conclusions drawn adequately supported by the results?"
  Partly as there would be good to see a discussion regarding the large gap between knowledge and practice - why do people not behave as they know they should? 

I have read this submission. I believe that I have an appropriate level of expertise to confirm that it is of an acceptable scientific standard.

# Response

**Published:** 2017-09-16

**Authors:** Trang Nguyen

**Affiliations:** Institute for Global Health Innovations, Duy Tan University, Vietnam

Thank you very much for your feedback. We have added more details in conclusion of abstract and main text, which referred to the high level of knowledge but low level of practice; and the experience might not always guarantee better practice. We have also removed a part of table 1 that were duplications.
